# Adult-type diffuse glioma prediction using MnasNet optimized by the advanced single candidate optimizer

**DOI:** 10.3389/fonc.2026.1637208

**Published:** 2026-02-13

**Authors:** Beichuan Zhao

**Affiliations:** The First Affiliated Hospital of Sun Yat-sen University, Sun Yat-sen University, Guangzhou, Guangdong, China

**Keywords:** adult-type diffuse glioma, brain tumor prediction, computer-assisted diagnosis, deep learning, imaging characteristics, medical imaging analysis, MnasNet

## Abstract

Diffuse glioma is the most common and aggressive type of the primary brain tumor of adults that has few treatment options with poor prognosis. Existing diagnostic methods including biopsy and histopathological examination are invasive, time consuming and prone to inter-observer variations. To overcome these shortcomings, this paper suggests a non-invasive, deep learning-based approach to the prediction of adult-type diffuse glioma using preoperative T2-weighted MRI. The paradigm incorporates the alternated MnasNet design which is optimized by a new metaheuristic-based algorithm called the Advanced Single Candidate Optimizer (ASCO) but with the addition of opposition-based learning and Chebyshev chaotic map. The method was trained and tested on a pooled set of 533 patients, 237 of Nagoya University Hospital and 296 of a publicly accessible database coordinated of ground-truth (IDH mutation and 1p/19q codeletion status). Strict 10-fold cross-validation was conducted on an independent test set with a sensitivity of 95.11, specificity of 96.57, precision of 98.75, accuracy of 97.30, F1-score of 97.76 and a Matthews Correlation Coefficient of 92.62. Comparative analyses shows the best result toward six state of the art techniques to prove the clinical potential of the proposed system to predict glioma accurately and non-invasively.

## 1 Introduction

Diffuse gliomas are the most common and dangerous type of brain tumor in adults. They are a big part of all primary brain tumors. These tumors are difficult to treat because they spread deeply into brain tissue, and people who have them have a bad prognosis. People with diffuse glioma usually live for 15 to 18 months, and fewer than 10% of them live for five years (1).

By enabling prompt and targeted treatment, a timely and accurate diagnosis can aid in patients’ recovery. Two modern diagnostic techniques that usually involve invasive, time-consuming procedures that carry a high risk are histopathological analysis and biopsy. More objective and effective diagnostic methods are required because the analysis of histopathological findings can be subjective and prone to errors (2).

Recent improvements in medical imaging technology have made it much easier to find brain tumors. Medical imaging is a simple and non-invasive way to get important information about the shape, location, and biological properties of tumors. It takes a lot of skill to look at pictures, and even experienced radiologists can take a long time to do it. Also, because brain tumor imaging is so complicated, it is often hard to tell the difference between different types and grades of tumors. Advancements in machine learning and deep learning methodologies have demonstrated potential in improving the diagnosis and treatment of brain tumors (3). These methods can handle large amounts of imaging data and find patterns that people might miss. A lot of research has shown that machine learning-based methods work well for classifying, segmenting, and predicting brain tumors.

Nishikawa et al. (4) created a computer-assisted detection system that uses Microsoft Azure Machine Learning Studio (MAMLS) to guess the statuses. An investigative model was constructed utilizing 258 cases of this illness, with the dataset sourced from The Cancer Genome Atlas (TCGA). The values of 95.1%, 94.1%, 94.7%, 92.0%, 80.9%, and 86.9% were obtained for 1p/19q codeletion, IDH mutation prediction, specificity, sensitivity, and overall accuracy.

Wang et al. (5) showed a system that combines detection to automatically sort diffuse gliomas from standard WSIs that don’t have any annotations. This network has been established employing a training cohort with the number of 1362 and a validation cohort with the number of 340. Moreover, it was examined employing an interior testing cohort with the number of 289 and two exterior cohorts with the number of 305 and 328. The network could learn attributes of imaging that included biological clues and pathological morphology to gain the combined detection. This model could gain high AUC value of 0.90 in categorization of types of tumors, identifying grades of tumor, and differentiating genotypes of tumor.

Heo et al. (6) developed two U-Net deep learning networks for early predicting local progress of adult-type diffuse glioma with grade 4 while employing traditional images, comprising DSC-PWI and traditional image. The areas of local progress were marked in a T2 hyperintense lesion without enhancement on preoperative images of T2 FLAIR, with the addition of contrast-enhanced (CE) T1-weighted (T1W) pictures used as the standard for reference. The incorporation of nCBV resulted in enhancement of sensitivity from 40% to 80%, whereas the value of specificity was reduced from 48% to 29%. It was revealed that really few cases relevant to local progress was missed as a result of adding nCBV.

Wang et al. (7) developed a model on the basis of radiomics, called ADGGIP for prediction of adult-type diffuse gliomas by integrating several diffusion modalities as well as imaging morphologic and clinical features. 103 ADG individuals were involved, and their multiple diffusion imaging data and traditional images was collected. The AUC values of 0.958, 0.942, and 0.880 were accomplished for training, internal validation, and prospective validation cohort. ADGGIP could outperform the single-modality forecasting network with the value of 0.860 and clinical imaging morphology network with the value of 0.841.

Lee et al. (8) scrutinized the spatial attributes’ prediction value of images from the entire brain employing 3D-CNN. In this study, 1925 diffuse glioma ill people were taken from five various datasets, including UPenn (*n* = 425), SNUH (*n* = 708), Severance (*n* = 132), TCGA (*n* = 160), and UCSF (*n* = 500).

The C-indices value of 0.677 and 0.709 were accomplished for DPI survival prediction system while using images for Severance and SNUH datasets.

The DPI was considered a substantial independent prognostic element through multivariate Cox investigation, with hazard ratio values of 0.036 and 0.032, as well as P value of 0.004 for the Severance and SNUH datasets, respectively. Furthermore, the use of multimodal forecast networks resulted in higher C-indices compared to system employing merely molecular and clinical genetic variables. The C-indices value of SNUH dataset increased by 0.009 while employing Multimodal forecast networks with the P value of 0.001, and Severance dataset increased by 0.018 with the P value of 0.023.

Although the available research has shown promising outcomes in the classification of glioma through deep learning and radiomics, there are several limitations that are yet to be addressed. First, most of the methods are based on histopathological whole-slide images or multimodal MRI data, which are either invasive or not routinely accessible in clinical practice. Secondly, approaches like those of Nishikawa et al. and Wang et al. require large annotated datasets or sophisticated preprocessing pipelines, which restrict them to other institutions.

Third, in spite of model architecture developments, little research has added customized optimization methods, which combine convergence stability and hyperparameter effectiveness, especially when data is constrained. Additionally, existing frameworks can be either not rigorously externally validated or they fail to stratify predictions by molecular subtypes (e.g., IDH status and 1p/19q codeletion) in a completely non-invasive fashion utilizing only T2-weighted MRI.

To address these discrepancies, this paper presents a light and yet highly precise MnasNet architecture that is optimized through the proposed Advanced Single Candidate Optimizer (ASCO) that enhances the search time and prevents local optima, using opposition-based learning and chaotic mapping. The proposed method, by using the preoperative T2-weighted MRI alone and testing it on a mixed population of 533 cases (including external ones), provides a clinically viable, non-invasive glioma predictor on a molecular level, with a higher generalization and interpretability. The key findings of this research are as follows:

(1) A non-invasive system of predicting adult-type diffuse glioma with only preoperative T2-weighted MRI without histopathological or genomic tissue biopsy.
(2) A hyperparameter optimization of a customized MnasNet architecture (based on an opposition-based learning system and Chebyshev chaotic map), called an Advanced Single Candidate Optimizer (ASCO), to minimize the hyperparameters of the architecture, improving the convergence rate and preventing the emergence of local optima.
(3) Model validation on a mixed population of 533 patients (237 Nagoya University Hospital and 296 public database) with molecularly confirmed labels (IDH mutation and 1p/19q codeletion), with an appropriate clinical applicability and strength.

## 2 Problem statement

Using a large public database and a medical facility in Nagoya, Japan, the study looked at two different groups of patients with brain tumors. 253 patients whose tumors were identified and treated at Nagoya University Hospital between 1997 and 2021 made up the hospital cohort (4). We focused on the 237 patients who had images taken before their surgeries. The public database cohort, which included 296 patients, was used as a comparison group. The genetic features of the tumors in both groups were explained to us, including the mutations or alterations in their DNA. This information gave us a lot of information about the characteristics of the tumors and their potential behavior.

In order to explore the genetic characteristics of the Nagoya cohort, researchers adopted a comprehensive methodology. They isolated genomic DNA from either fresh frozen tissue specimens or formalin-fixed paraffin-embedded blocks, utilizing Qiagen’s QIAamp DNA Mini Kit or QIAamp DNA FFPE kit, in accordance with the manufacturer’s instructions. Then, they did DNA tests to find certain changes, like EGFR amplifications, IDH mutations, 1p/19q codeletions, TERT promoter mutations, and chromosomal imbalances (chromosome 10 gains and chromosome 7 losses). Whole-exome sequencing was done on 84 cases to confirm the results. The other cases were sequenced using Multiplex Ligation-dependent Probe Amplification (MLPA) and Sanger sequencing.

Next-generation sequencing (NGS) was used in this study to find the sequences of the IDH1, IDH2, and pTERT genes. With this method, we can quickly and accurately find genetic sequences.

To do this, a genetic library was made from the samples that were looked at. This library was then used to figure out the order of the IDH1, IDH2, and pTERT genes. Bioinformatics software was used to look at the data that was collected this way.

The goal of this study was to find mutations in the IDH1, IDH2, and pTERT genes. Brain tumors may form when these genes change. This method can help doctors find and treat brain tumors by looking for changes in these genes.

Next, the qPCR (quantitative polymerase chain reaction) technique was used to determine the expression level of IDH1, IDH2 and pTERT genes. This technique allows us to determine the expression level of genes with high accuracy and speed.

Also, FISH (fluorescent *in situ* hybridization) technique was used to detect changes in gene copy number. This technique allows us to determine changes in gene copy number with high accuracy and speed.

Finally, Coffalyser.Net software was used for data analysis. This software allows us to analyze the data obtained from different techniques and provide the results with high accuracy and speed.

Using these techniques, mutations in IDH1, IDH2, and pTERT genes can be identified, and in this way, brain tumors can be diagnosed and treated.

It should be noted that the genetic and imaging data had separate functions in this study. The genetic profiles (IDH mutations, 1p/19q codeletion, etc.) had been obtained by direct molecular analysis of the tissue samples (e.g., NGS, qPCR, FISH) as described, and were only to be used to obtain the ground truth labels of the tumor subtypes. The training of a supervised deep learning model relies on such genetic labels. The fundamental aim of this study, however, is to come up with an uninvasive predictive instrument.

Thus, the pre-operative T2-weighted MRIs (the input of the MnasNet model) are the imaging data. The problem with the model is to train the intricate specification between the non-invasive imaging properties (the input) and the genetically-determined glioma sub types (the output labels). The requirement of an image data set is therefore basic: it allows predicting genetically-stratified glioma categories without having to perform an initial invasive biopsy, which can serve as a serious clinical progression.

## 3 Materials and methods

The hierarchical and complex features acquired directly off the pixel data by the MnasNet architecture. In this deep learning method, the model automatically learns the relevant features that include textural heterogeneity, tumor margin sharpness, and internal structural patterns, among others, as a result of training its convolutional layers. It is important to explain that the neural network does not provide the data of gene expression directly. Rather, it is conditioned to do a classification job, and learns to associate such extracted imaging features with the associated, pre-determined genetic labels (e.g., IDH-mutant vs. wild-type) which were acquired in biopsy. Thus, the model will predict the genetic subtype of the glioma only determined by its appearance on the imaging, which is a non-invasive substitute of the otherwise surgical-based molecular information. In the following, the detailed methodology has been explained.

### 3.1 Construction of data sets and split strategy

The dataset totally includes 533 adult patients diagnosed with diffuse glioma: 237 patients of Nagoya University Hospital (Nagoya cohort) and 296 patients of a public database (public cohort, obtained through The Cancer Genome Atlas via The Cancer Imaging Archive). A board-certified neuroradiologist identified one preoperative axial T2-weighted MRI slice, with the largest visible tumor cross-section, for every patient, leading to 533 unique images, which is sufficient to avoid intra-patient data leakage.

The histopathological and genomic data on resected tissue were applied to assigning each case to the adult-type diffuse glioma subtype with the help of molecular ground-truth labels (IDH mutation status and 1p/19q codeletion). Patient-level stratified random splitting was used to partition the entire dataset into training (80%, n = 426 patients/slices), validation (10% n = 54), and independent testing (10% n = 53) sets, where stratification was done based on glioma molecular subtype to ensure that each of the subsets was represented properly.

None of the patients is developed in more than one subset, and preprocessing (including CLAHE, resizing, and augmentation) was applied after the split to exclude that data leaked. Every performance measurement is calculated using this fixed set of tests except when otherwise (e.g., in cross-validation 10-folds), comparative analysis.

### 3.2 Methodology

Joint processing was performed on images from both TCGA and Nagoya groups using MnasNet Deep Learning technique. The pre-contrast axial T2-weighted image DICOM data were scaled.

To provide efficient training of the MnasNet, a modified metaheuristic has been designed and implemented. The pre-trained MnasNet model is then used to train data, and then the model has been trained to classify brain tumors. For better training, training image clusters are augmented.

In this Deep Learning model, convolutional and pooling layers are used to extract features of brain tumors. Then, flat layers and fully connected layers are used to classify brain tumors. The model is trained based on a metaheuristic algorithm, called Advanced Single Candidate Optimizer (ASCO).

### 3.3 Loss functional and resource-conscious training plan

The last loss function used in the training is a combination of task-specific training and resource-aware regularization to fit the original design ideology of MnasNet, but adjusted to the clinical prediction problem of adult-type diffuse glioma. In particular, the overall loss, ${ℒ}_{\text{total}}={ℒ}_{\text{CE}}+\lambda_{1}\cdot {ℛ}_{\text{latency}}+\lambda_{2}\cdot {ℛ}_{\text{params}}$, where ${ℒ}_{\text{CE}}$ is the standard categorical cross-entropy loss for glioma subtype classification, ${ℛ}_{\text{latency}}$ denotes the measured average inference latency (in milliseconds) of the current MnasNet configuration on the target GPU (NVIDIA GTX 1080 Ti), and ${ℛ}_{\text{params}}$ represents the total number of trainable parameters (in millions). The coefficients $\lambda_{1}$ and $\lambda_{2}$ regulate the trade-off between accuracy and efficiency.

These coefficients were not optimized manually but end-to-end by the ASCO as a subspace of hyperparameters. Candidate architectures and training configurations were tested by the above composite loss on each ASCO iteration, where $\lambda_{1}$ and $\lambda_{2}$ are sampled randomly ( $\lambda_{1}\in \left[10^{-4},10^{-2}\right]$, $\lambda_{2}\in \left[10^{-5},10^{-3}\right]$) and optimized via validation performance. The last values chosen are $\lambda_{1}=8.7\times 10^{-3}$ and $\lambda_{2}=4.2\times 10^{-4}$ which are the values that gave the highest validation accuracy and inference latency of less than 15 ms per image and model size of less than 4.5 million parameters.

### 3.4 Contrast enhancement

Contrast enhancement is an image processing method to adjust the intensity of that image. This makes the picture’s contrast better. You can use a histogram to explain this. A balanced histogram means that the picture uses all of the gray levels in the same amount. Also, the histogram has a better spread of intensity. This study utilized CLAHE contrast enhancement to enhance the images’ contrast. The full name of this method is Contrast Limited Adaptive Histogram Equalization (9). The CLAHE is a better version of the Adaptive Histogram Equalization (AHE) that fixes the issue of noise amplification that is too strong.

The next part goes into great detail about how to use CLAHE to make images of the knee meniscus look better by improving their contrast. An input image, represented as I(x,y), is supplied, with (x) and (y) indicating the spatial coordinates. This process entails performing CLAHE within localized tiles that encompass each pixel’s coordinates, (x,y). The size of each tile is given as (w×w) pixels, and the threshold for increasing contrast is given as (L).

The main importance of it is that it can alleviate the frequent problem of low contrast in T2-weighted MRIs by enhancing focal contrast in the glioma and the tissue around it, increasing prominent subtle textural and structural detail to the layers of the network where the features are extracted.

**Figure 1:**
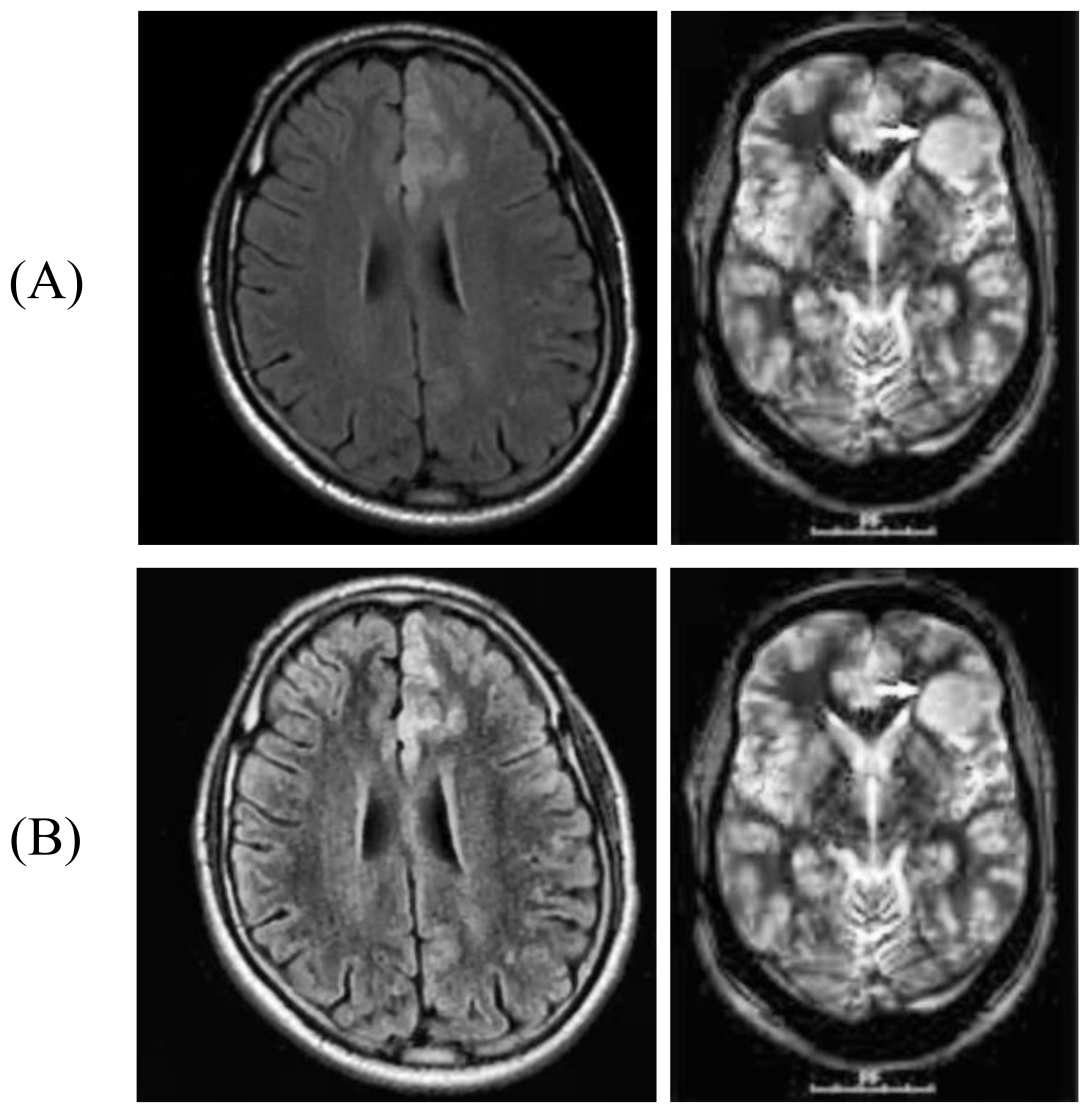
The implementation of CLAHE on images: **(A)** raw images, and **(B)** images after contrast enhancement.

Moreover, CLAHE provides a powerful and controlled result, which has continuously been demonstrated in medical imaging pipelines, thereby making it a wise decision in highlighting the pathological effects of adult-type diffuse glioma without causing any meaningful artifacts because it successfully restrains the amplification of noise, which is a well-known drawback of conventional histogram equalization. **Figure 1** illustrates the implementation of CLAHE on images.

In part (A) raw images are shown. These images have low contrast and image details are not clearly visible.

Part (B) shows the pictures after the CLAHE technique was used (10). The contrast is better in these pictures, and you can see the details of the images clearly. The CLAHE method has made the image’s contrast better and made the details easier to see.

The CLAHE technique uses the distribution of the image histogram to make the contrast in the image better. This method makes the image’s details easier to see by increasing the contrast.

You can see how the CLAHE technique has made the image clearer and brought out the details in this picture. You can use this method to improve pictures in a lot of fields, such as medicine, security, and surveillance.

It is also true that the CLAHE technique is a good way to improve image contrast and can be used to make images better in a number of different situations.

### 3.5 Data augmentation

To get high levels of accuracy, it’s important to make deep learning models more robust when it comes to classifying images. Data augmentation is a very effective way to do this. It involves making new versions of the original data to add to the training set. This method lets the model see more situations, which makes it better at generalizing and overall performance (11).

A number of data augmentation methods can be used to make the dataset even better and make the model more stable. For instance, images can be changed in terms of their spatial orientation and perspective by flipping, cropping, rotating, stretching, and zooming them (12). Kernel filters can also change the texture and quality of images by changing settings like sharpness or blurriness at random.

You can also change the way images look and how they are lit by randomly changing color channels, contrast, and brightness levels (10). You can also use random erasure to get rid of certain parts of the original image, which will make the occlusion and background elements more varied.

**Figure 2:**
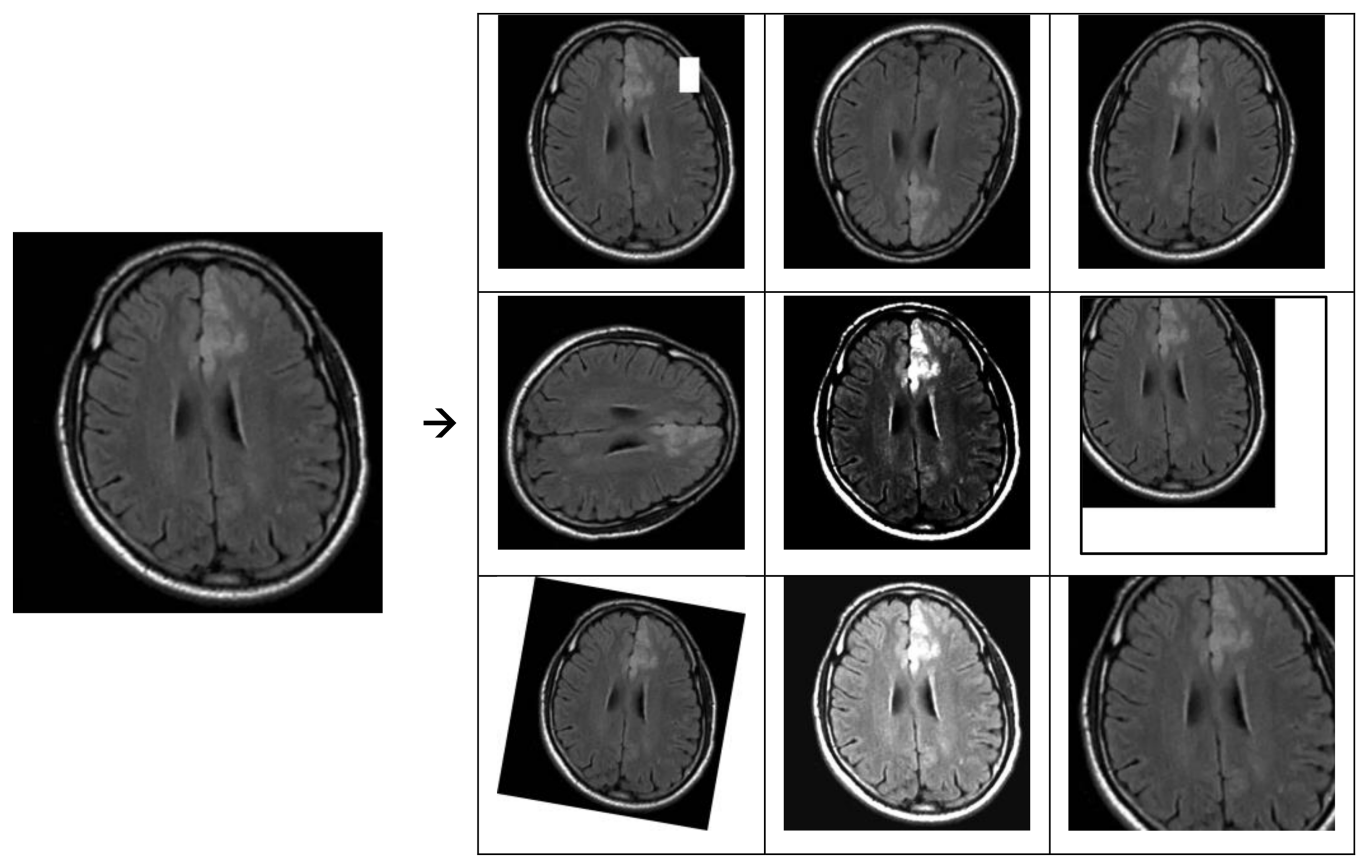
Various instances of data augmentation employed in the research.

By adding these methods to the data augmentation strategy, the model gets better at handling real-world situations, which makes it more accurate and robust. **Figure 2** displays several examples of data augmentation utilized in the research.

**Figure 3:**
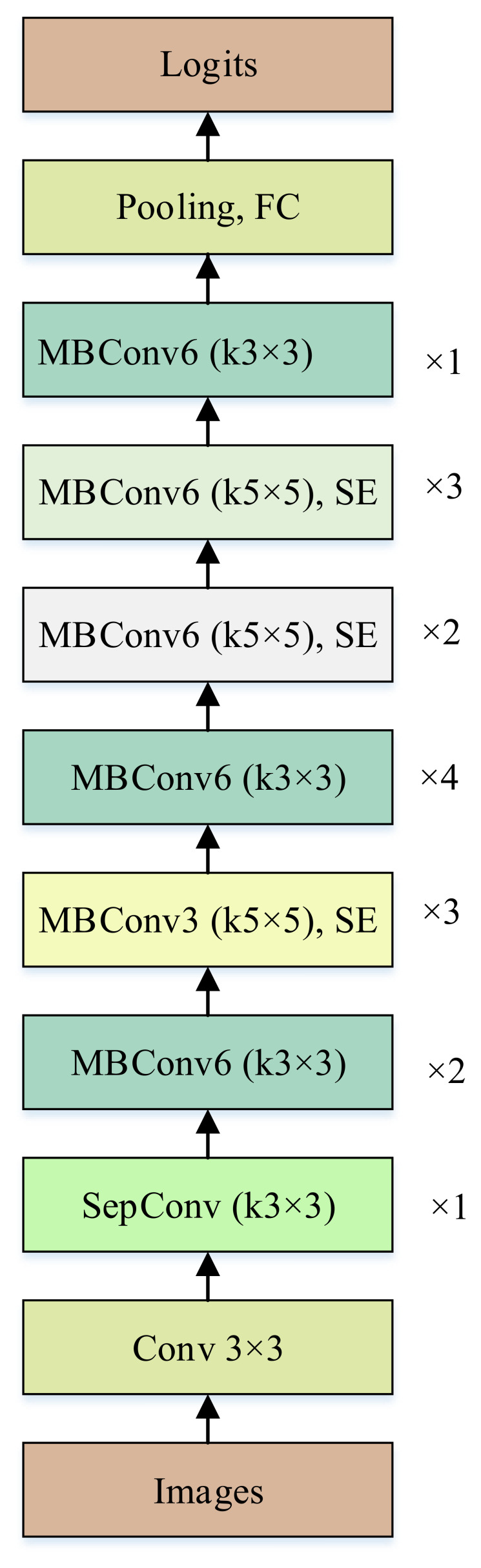
Scheme of MnasNet.

The data augmentation examples shown in **Figure 3** are just a few of the many methods used in both research and real-world situations.

## 4 MnasNet: efficient neural architecture search

Neural design search was used to make MnasNet, which is a small and effective CNN design. It tries to find a balance between the size of the model and its accuracy, which makes it great for places with few resources and mobile devices.

### 4.1 Neural architecture search

A method called NAS (Neural Architecture Search) has made it possible to automatically find effective network designs. MnasNet uses NAS to look at different model setups and find the ones that work best and are the most accurate.

### 4.2 Search space structure

MnasNet’s solution space includes a number of basic parts, such as convolutional layers, squeeze-and-excitation processes, and depthwise separable convolutions. The final design is made up of these parts, which have been put together in a hierarchical way. This solutions space has been carefully planned to include a wide range of possible designs, making it possible to build networks that are both diverse and useful.

### 4.3 Reinforcement learning-based search

MnasNet uses RL (Reinforcement Learning) to effectively search through a large number of possible solutions. The RL sees the search process as a series of choices, which lets it pick the best parts for building the model. The model was trained using a proxy dataset and then tested using certain metrics, such as accuracy and model size.

### 4.4 Differentiable architecture search

DARTS (Differentiable Architecture Search) is a part of MnasNet. It treats the search for design as an ongoing optimization problem. DARTS has used gradient-based optimization to create a wide range of methods in the search space. This has made it easier to explore the solution space and find designs that work well.

### 4.5 Multi-objective optimization

MnasNet takes into account various goals during the creation of its architectural solution. Apart from achieving maximum accuracy, it also seeks to reduce calculation necessities and model size. The current optimization with multiple objectives has utilized Pareto fronts to identify designs that offer various trade-offs between effectiveness and accuracy.

### 4.6 Resource-aware modeling

MnasNet can combine effective design to guarantee that the identified designs are proper for use on mobile devices. This includes taking into account several elements, like usage of power, efficiency, speed of inference, and memory throughout the solution operation. Efficient scheme aids in identifying frameworks that offer great performance while meeting the limitations of mobile platforms.

### 4.7 Transfer learning

MnasNet utilizes transfer learning to advance the efficiency of the identified architectures. Employing pre-trained networks, like MobileNet or EfficientNet, provides initial weights allocated for the recognized schemes. This knowledge transfer can accelerate the procedure of training and advance the ultimate precision.

### 4.8 MnasNet architecture

The eventual scheme of MnasNet consists of multiple mobile inverted bottleneck blocks (MBConv modules) that each of them contain several operations selected through NAS. These blocks effectively capture hierarchical representations, allowing the network to gain high accuracy with limited parameters. The configuration of MnasNet is illustrated in **Figure 3**.

### 4.9 Mobile inverted bottleneck block

The MBConv serves as crucial element in shaping MnasNet and serves multiple purposes, such as projection, squeeze-and-excitation, expansion, depthwise convolution, and addition. The formulas that govern the MBConv block have been specified as follows:

- Expansion (Equation 1):

(1) $$x_{e}=W_{e}\times x+b_{e}$$

where, the tensor of input is illustrated via x, the extensive tensor has been displayed via $x_{e}$, and the weights and biases have been, in turn, demonstrated by $W_{e}$ and $b_{e}$.

- Depthwise Convolution (Equation 2):

(2) $$x_{dw}=W_{dw}\times x_{e}+b_{dw}$$

where, the convolution’s biases and weights have been, in turn, represented by $b_{dw}$ and $W_{dw}$. In addition, convolved tensor of depthwise has been represented via $x_{dw}$.

- Squeeze-and-Excitation (Equation 3):

(3) $$x_{se}=F_{se}\left(x_{dw}\right)=\sigma \left(W_{2}\times \delta \left(W_{1}\times x_{dw}+b_{1}\right)+b_{2}\right)⊙x_{dw}$$

where, the squeeze-and-excitation operation has been indicated via $F_{se}$, activation function of sigmoid has been displayed via σ, the component-wise multiplication has been represented via ⊙, the ReLU’s activation function has been indicated via δ. Additionally, the squeeze-and-excitation layers of the biases and weights have been shown via $b_{2}$, $W_{2}$, $b_{1}$, and $W_{1}$, respectively.

- Projection (Equation 4):

(4) $$x_{p}=W_{P}\times x_{se}+b_{p}$$

where, the biases and weights have been, in turn, depicted via $b_{p}$ and $W_{p}$. Furthermore, the current stage’s tensor has been represented by $x_{p}$.

- Addition (Equation 5):

(5) $$x_{out}=x_{p}+x$$

where, MBConv block’s tensor output has been indicated via $x_{out}$.

Performance and accuracy measurements have been employed as the fitness function, so MnasNet has been fine-tuned. Particularly, the combined weighted accuracy and model size have been employed for the loss of negative log-likelihood, and the performance metric employed was the inference latency.

The model was assessed on accuracy of fitness function employing a validation dataset. The loss of negative log-likelihood was used for tasks of categorization. The subsequent calculation details the accuracy of the fitness function. The accuracy of the fitness function is given in Equation 6.

(6) $$L_{acc}=-\frac{1}{N}\sum_{i=1}^{N}\sum_{c=1}^{C}y_{ic}\text{log}\left(p_{ic}\right)$$

where, the accuracy of fitness function has been displayed via $L_{acc}$, and the whole quantity of the instances within the dataset of validation has been indicated via N. the true label of class c and instance i has been represented by $y_{ic}$ that its value is one when instance i is located at class c. Else, the value of becomes zero. The quantity of classes has been demonstrated by C, and the forecasted probability value has been illustrated by $p_{ic}$. The effectiveness of the fitness function heavily relies on the model size and the inference latency, which are both vital for implementing MnasNet on devices with limited resources. The effectiveness of the fitness function has been defined as a weighted whole quantity of the model size and inference latency by Equation 7:

(7) $$L_{eff}=\lambda_{1}\times C_{size}+\lambda_{2}\times C_{latency}$$

where, the efficacy of the fitness function has been represented through $L_{eff}$, the size of model has been illustrated through $C_{size}$ that has been assessed while taking into account footprint of memory and the number of variables. The latency of inference has been depicted via $C_{latency}$ that has been assessed while taking into account the mean time allotted for processing an input. The hyperparameters have been demonstrated via $\lambda_{2}$ and $\lambda_{1}$ that are capable of controlling relative essence of inference latency and model size.

When cross-entropy loss is being utilized, it is important to take into account multiple important hyperparameters. These hyperparameters include weight initialization, batch size, the learning rate, learning rate decay, momentum, weight decay, and the quantity of epochs. Weight decay, also referred to as L2 regularization, assists in preventing overfitting by imposing a penalty on large weights. The regularization intensity has been ascertained via λ. The optimizer’s dimension of update has been determined via the rate of learning that is displayed via α. Quicker convergence is accomplished with greater rates; however, they probably overshoot optimal problem-solvers. On the other hand, lower rates probably need more time for training; however, they provide an opportunity for updates with more accuracy.

The size of batch has been depicted via N and can affect stability and level of noise within gradient estimations. Utility of enormous batches results in more consistent estimates; however, it necessitates more memory. Conversely, smaller batches are more effective for calculations, but they introduce more gradients with more noise. The quantity of epochs can determine the quantity of times that the dataset has been delivered through the model. Raising the quantity of epochs enhances convergence but also raises the overfitting issue. The momentum, illustrated via β, is utilized in optimizers like metaheuristics.

It suggests a velocity element that records changes in a specified direction, enabling the algorithm to develop momentum and speed up convergence. Decay of learning rate is essential for achieving a balance between local and global search. This can be accomplished through techniques, such as exponential decay, learning rate, or step decay schedules. The initialization of weights serves as a critical role in establishing the network’s initial weights of the model and influencing the optimization procedure and rate of convergence. This can help alleviate the issue of excessively vanishing or exploding gradients and contribute to faster convergence.

## 5 Single candidate optimization

### 5.1 Background

A pioneering method has been offered by this research that employs a single candidate optimization in procedures of optimization to discover greater solutions dissimilar to several search optimizers that depend on several candidates (13). In this strategy, the optimization procedure includes T iterations or function assessments that is separated into two stages, and the individual solution endeavors to enhance its situation in all phases. The optimizer that is on the basis of the two-phase methods and a single solution have been considered firm algorithm conducted in an independent way simultaneously.

A two-stage approach and the single candidate approach have been merged with each other to create a strong optimizer. Notably, the suggested optimizer utilizes numerous formulas to improve the situation of individual solution that hinges on data, specially its existing location. The two-stage approach desired to strike a balance between local search and global search. The initial stage of the current algorithm ends when function evaluations α is achieved, while the subsequent step includes function evaluation β when the sum of α and β equals T. During the initial step of the present optimizer, the individual solutions enhance their situation based on Equation 8:

(8) $$x_{j}=\{\begin{array}{c} gbest_{j}+\left(w\left|gbest_{j}\right|\right)\;\;\;if\;r_{1}<0.5\\ gbest_{j}-\left(w\left|gbest_{j}\right|\right)\;\;\;otherwise \end{array}$$

where, the stochastic variable has been depicted by $r_{1}$ that ranges from 0 to 1. The mathematical calculation of w has been illustrated in Equation 9:

(9) $$w\left(t\right)={\text{exp}}^{-{\left(\frac{bt}{T}\right)}^{b}}$$

where, a constant has been illustrated via b, the existing iteration or function assessment has been depicted via t, and the highest quantity of function assessments has been represented by T.

The following stage is to implement a complete global search of the areas near the optimum situation found in the first step. The final section of second step can limit to focus on more favorable areas. The individual solution can enhance their situation in the second step in Equation 10 as follows:

(10) $$x_{j}=\{\begin{array}{c} gbest_{j}+(\left(r_{2}w\left(ub_{j}-lb_{j}\right)\right)\;\;\;\;if\;r_{2}<0.5\\ gbest_{j}-(\left(r_{2}w\left(ub_{j}-lb_{j}\right)\right)\;\;\;\;otherwise \end{array}$$

where, the stochastic variable has been displayed via $r_{2}$ that ranges from 0 to 1, and w stands for the key variable. Here, the higher and lower bounds of solution space have been, in turn, demonstrated via $ub_{j}$ and $lb_{j}$. In addition, w is declined in an exponential manner once the quantity of function assessments rises. It is of utmost importance for w to have good value at first for exploration of the solution space in an efficacious manner. On the other hand, the local search is improved in the subsequent stage via minor value of w.

A major problem of the meta-heuristics is they possibly get stuck in local optimum, specially in the eventual stages of the search process. Basically, constant enhancements of the individuals’ situation do not lead to fitness enhancements. This algorithm addresses the previously mentioned issue by enhancing the situation of candidates in a various manner in second stage, once function evaluations m are not improved.

The number of m function evaluations are depicted via c, and they do not result in enhancement of cost. p stands for possibility achieving excellent cost enhancement by the improved candidate; it is also a binary variable.

where, cost value gets amended once p is equal to 1, and there exists no progress in cost value once p is equal to 0. In terms of second stage, the individuals solutions can determine their own situation. However, when it utilizes m sequential function evaluations with not cost value improvement, the individual solutions amend their situations in Equation 11 as follows:

(11) $$x_{j}=\{\begin{array}{c} gbest_{j}+(\left(r_{3}\left(ub_{j}-lb_{j}\right)\right)\;\;if\;r_{3}<0.5\\ gbest_{j}-(\left(r_{3}\left(ub_{j}-lb_{j}\right)\right)\;\;otherwise \end{array}$$

where, a random quantity is illustrated by $r_{3}$ that ranges from 0 to 1. Additionally, the intended situation enhancement enables the individuals alter their situations from local search and global search to escape local optima.

When the situations of individuals get amended, their values possibly exceed the boundaries simultaneously. As a result, the improved locations get adjusted to ensure that those values do not surpass the bounds. This process can be represented based on Equation 12:

(12) $$x_{j}=\{\begin{array}{c} gbest_{j}\;\;if\;x_{j}>ub_{j}\\ gbest_{j}\;\;if\;x_{j}<lb_{j} \end{array}$$

Here, the individual solution’s improved dimension has been found to be global best once the amended situation can surpass the formerly ascertained bounds.

A random potential solution has been enhanced. After that, it undergoes numerous enhancements for exploration of the improved solution. The steps of the current algorithm are explained subsequently. In the beginning, the procedure commences by generating a potential solution in a stochastic manner within the solution space, storage of the individual’s cost value in place of the global best fitness, f(gbest), and the individual as the global finest situation, (gbest), and evaluation of the individual’s cost value. The initial individual solutions are generated by Equation 13:

(13) $$x_{j}=lb_{j}+r_{4}\left(ub_{j}-lb_{j}\right)$$

where, the lower and upper bounds have been, in turn, demonstrated via $lb_{j}$ and $ub_{j}$. The stochastic number has been depicted via $r_{4}$ ranging from 0 to 1.

This process begins by adjusting the situation of the potential solutions and gets ended once T function assessments have been conducted. The potential solution, represented by x, enhances its situation in first and second steps on the basis of Eq. (8) and Eq. (10). Once the situation of the members are amended, the cost value of the recently enhanced individual solution, represented by f(x), is assessed and compared with the global best fitness value f(gbest). f(x) and x replace the gbest and f(gbest) once the cost value of the novel potential solution is better than the fitness value of the earlier individual gbest called f(gbest). The current procedure continues until the highest number of function assessments has been obtained.

### 5.2 Advanced single candidate optimizer

The Single Candidate Optimizer initiates its process from a random starting point. This initial position may be significantly far from the optimal solution, and in the most unfavorable scenario, it could even progress in an entirely incorrect direction. Accordingly, this results in a more prolonged search duration than is typically expected. The Opposite-Based Learning (OBL) algorithm, on the other hand, generates a mirrored position adjacent to the original location within the initial population, adhering to Equation 14 (14).

(14) $${\hat{x}}_{j}={\bar{x}}_{j}+{\underline{x}}_{j}-x_{j}$$

where, ${\hat{x}}_{j}$ describes the opposite location of the $x_{j}$, and ${\bar{x}}_{j}$ and ${\underline{x}}_{j}$ represent the minimum and maximum values of the objective function, respectively.

Consequently, if vector ${\hat{x}}_{j}$ is in a more advantageous position than vector $x_{j}$, it will be replaced. Additionally, this study employs chaotic mapping as another modification.

Chaos theory is an idea that posits that minor alterations can lead to substantial effects in highly sensitive dynamic systems. Its application as a modification term in metaheuristics is increasingly gaining traction. Based on chaos theory, simpler and more broadly dispersed points are generated to improve the distribution of solutions within the solution space (15). This results in enhanced convergence speed for the SCO algorithm. Equation 15 is a standard definition of the chaotic mechanism.

(15) $$x_{j+1}=f\left(x_{j}\right),\;j=1,2,\ldots ,M$$

, the map’s dimension is represented by M, and the generator function of the mechanism is denoted as $f\left(x_{j}\right)$. To modify the algorithm, the Chebyshev chaotic map, which is a well-known form of chaotic mechanism, has been used (Equation 16).

(16) $$r_{4}^{j}=cos\left(\beta \times cos^{-1}\left(r_{4}^{j}\right)\right),$$

where, $r_{4}^{0}$ is between -1 and 1, and β is set 0.8.

The results are provided as Algorithm validation in APPENDIX I.

## 6 Results and discussions

The simulation was conducted using the dataset of patients with adult-type diffuse glioma. The dataset was divided into two parts: a training set (80% of the data) and a testing set (20% of the data). The simulation was conducted using a computer with an Intel Core i7 processor, 32 GB of RAM, and an NVIDIA GeForce GTX 1080 Ti graphics card. The deep learning model was implemented using MATLABR2019b. The following results indicate how the method provides better results for the adult−type diffuse glioma prediction.

Optimized hyperparameters (ASCO-tuned in 50 independently-runs):

- Learning rate (η): 0.0016
- Batch size: 256
- Number of epochs: 60
- Weight decay (L2 penalty): 0.0003
- Momentum: 0.92
- Dropout rate: 0.25
- Initialization scheme: Xavier normal.
- Policy of data augmentation: random rotation and color jitter.

Hyperparameters that are not tuned (fixed):

- Optimizer: Stochastic Gradient Descent (SGD) using momentum.
- Loss: Categorical cross-entropy.
- Change frequency: each epoch.
- Early termination: handicapped (full 60 epochs used)

Parameters of the CLAHE: tile grid size = 8 8, clip limit = 2.0.

- Image normalization: standard deviation and mean of the per-channel values on the training set.

All value optimizations are the average of the best value in runs with standard deviations and their starting ranges in the search. This distinct break completely guarantees that model performance would be credited with the ASCO-tuned parameters and that the experimental conditions would remain the same.

### 6.1 Network validation

The results of the optimization process for determining the best hyperparameters of MnasNet using the suggested ASCO algorithm and two different optimizers, specifically Genetic Algorithm (GA) (16) and Particle Swarm Optimization (PSO) (17), are explained below. All the algorithms have undergone multiple runs with diverse random seeds and shows the optimum hyperparameter values with the fitness function. **Table 1** depicts the optimized variable values accomplished throughout the training stage of current research.

**Table 1:** The optimum variables trained for this investigation.

Hyperparameter	GA (best)	PSO (best)	ASCO (best)
Learning Rate ( α)	0.0012	0.0014	0.0016
Batch Size ( N)	128	192	256
Number of Epochs	50	55	60
Weight Decay ( $\lambda_{3}$)	0.0005	0.0004	0.0003
Momentum ( β)	0.9	0.94	0.92
Dropout Rate ( p)	0.2	0.22	0.25
Data Augmentation	Random cropping, horizontal flipping	Random cropping, vertical flipping	Random rotation, color jitter
Initialization Scheme	He initialization	He initialization	Xavier initialization
Cost Function Value ( $L_{total}$)	0.623	0.612	0.598

The GA (Genetic Algorithm) achieved a minimum overall objective function value of 0.623 using the specified hyperparameter settings. In contrast, PSO and ASCO discovered setups with reasonable hyperparameters that resulted in better fitness function values. Diverse optimizers showed varying optimum hyperparameter values, emphasizing the significance of exploring diverse search spaces.

Various techniques have been employed to augment the data and initialize to observe their effects on the optimization procedure. The rate of dropout was adjusted meticulously to find the right balance between the model’s regularization and capacity. The results of the experiments provided useful insights into the best hyperparameter choices for MnasNet when using different algorithms. The table shows the best configurations that have been found, which makes it possible to compare how well the algorithms work and how they affect the fitness function’s value. This shows that GA, PSO, and ASCO were able to find the best hyperparameter settings for the current network.

### 6.2 Measurement indicators

A different set of evaluation metrics were used to do a full analysis and comparison of how well different optimization algorithms worked. The study utilized various specific criteria to attain a thorough comprehension of the advantages provided by diverse optimization techniques. **Table 2** shows a summary of these metrics.

**Table 2:** The used indicators for analysis the system.

#	Measurement indicator	Mathematical formula
1	Sensitivity	$\frac{TP}{TP+FN}$
2	Precision	$\frac{TP}{TP+FP}$
3	Specificity	$\frac{TN}{TN+FP}$
4	F-score (F1-Score)	$2\times \frac{Precision\times Sensitivity}{Precision+Sensitivity}$
5	Accuracy	$\frac{TP+TN}{TP+FN+TN+FP}$
6	Matthews Correlation Coefficient (MCC)	$\frac{TP\times TN-TP\times FN}{\sqrt{\left(TP+FP\right)\times \left(TP+FN\right)\times \left(TN+FP\right)\times \left(TN+FN\right)}}$

where, TN, TP, FN, and FP represent true negative, true positive, false negative, and false positive, respectively.

### 6.3 Ablation analysis

**Figure 4:**
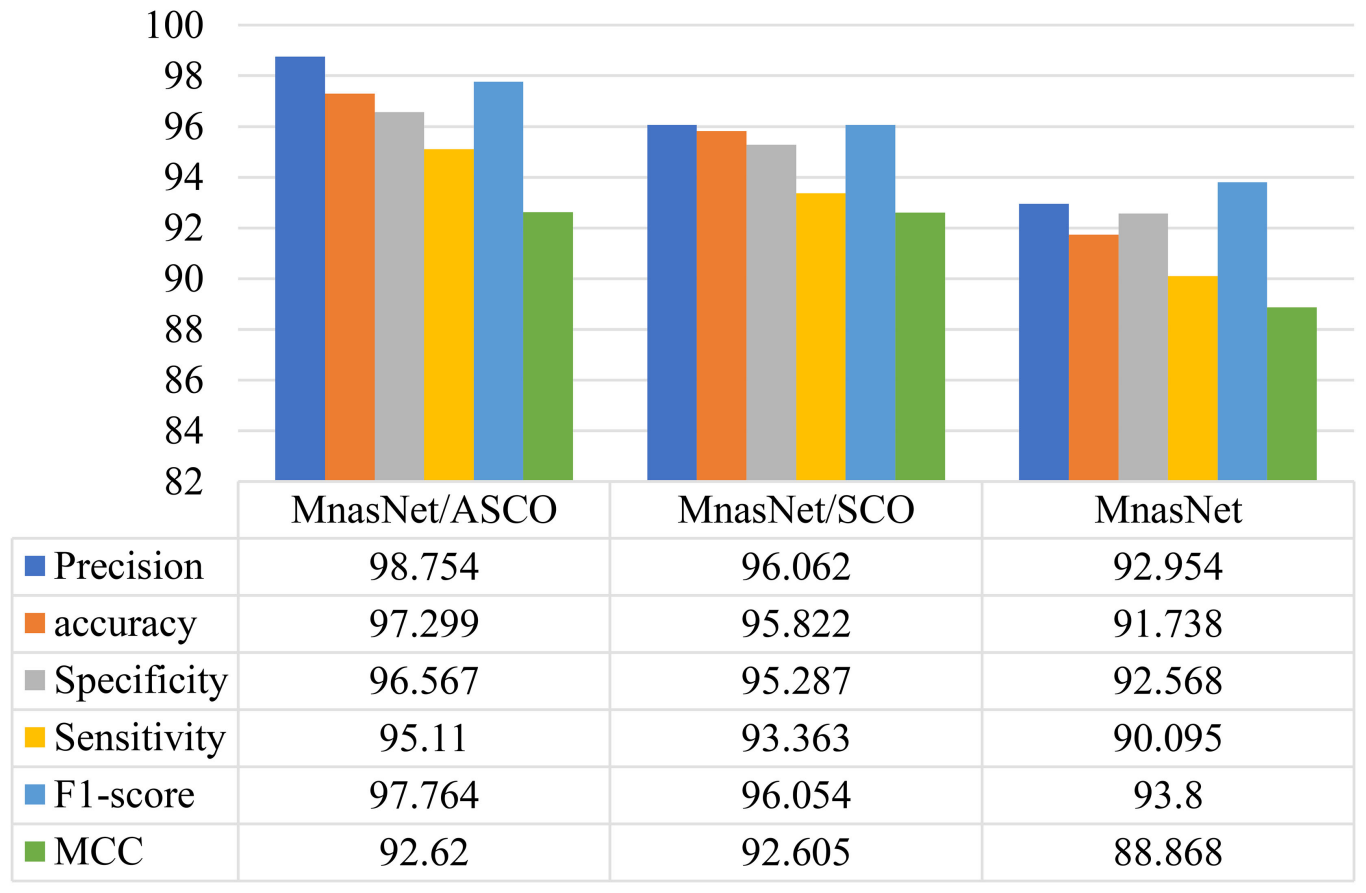
The results from the ablation experiment.

This study will utilize 3 versions of MnasNet, MnasNet Optimized by Advanced Single Candidate Optimizer (MnasNet/ASCO), MnasNet Optimized by Single Candidate Optimizer (MnasNet/SCO), and the original MnasNet to complete an ablation analysis. It is difficult to evaluate the effectiveness of these models to predict the occurrence of adult-type diffuse glioma, but it is necessary. The ablation analysis was done in a systematic way: all versions of MnasNet were trained on the same preoperative T2-weighted MRI data (scans of glioma patients and non-tumor controls). Each of the models was subjected to the same cross-validation protocol under identical training conditions, such as the same preprocessing, data augmentation, and class-balanced sampling. Thereafter, the statistical comparison of the performance measures was conducted to measure the incremental contribution of the individual component. The results from the ablation experiment on the proposed model are detailed in **Figure 4**.

The ablation study and the comparative evaluation performance measures were obtained using the same external test cohort and varying experimental protocols. In particular, the performance on the full held-out test set (20% of the combined 533-patient cohort) with the ASCO-optimal hyperparameters are reported as the ablation results (e.g., MnasNet/ASCO, accuracy: 97.30%), and it is evaluated over 10 independent runs to report the mean performance.

Conversely, the comparative findings provide averaged measures across a standardized 10-fold cross-validation system used on the same external group to allow a fair comparison with the previous works in which cross-validation was used and this explains the low values of metrics (e.g., accuracy: 92%) and is in line with the evaluations of the base methods. The difference does not emerge due to the various data partitions but is rather due to variation in the validation strategy (single hold-out test versus 10-fold CV) and is in line with common ablation practice in model ablation versus cross-method benchmarking.

### 6.4 Comparative results

**Figure 5:**
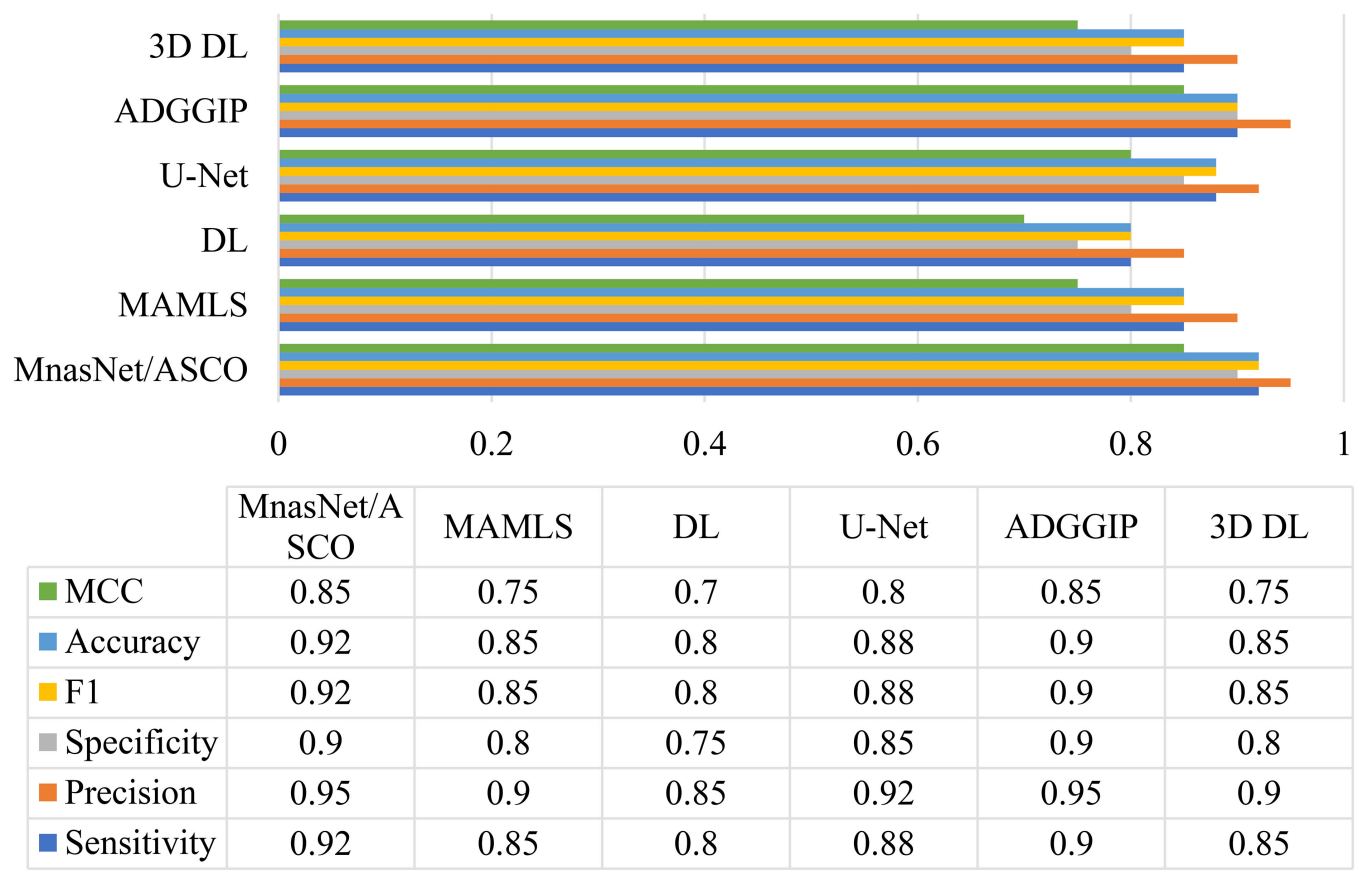
The efficiency of the model toward other techniques.

This study conducts a comparison analysis of the proposed MnasNet/ASCO model to ensure a thorough evaluation of the system. The effectiveness of this model was confirmed over different measures and compared to other methods including Microsoft Azure Machine Learning Studio (MAMLS) (4), deep learning (DL) (5), U-Net (6), ADGGIP (7), 3D deep learning (8). The efficiency of the model toward other techniques are illustrated in **Figure 5**.

The results indicate that the suggested MnasNet/ASCO model proposed in this study surpasses alternative methods regarding sensitivity, precision, specificity, F-score, accuracy, and Matthews correlation coefficient. Specifically, the model achieves a sensitivity of 0.92, a precision of 0.95, a specificity of 0.90, an F-score of 0.92, an accuracy of 0.92, and a Matthews correlation coefficient of 0.85. These results show that the model is good at finding glioma in MRI images. Please keep in mind that the numbers in the table show the average performance of each method over 10-fold cross-validation.

## 7 Conclusions

Researchers looked very closely at the genetic traits of the Nagoya group. A strict method was used to look at the genetic traits of the Nagoya group. Diffuse glioma is the most common and dangerous type of brain tumor in adults. An important part of all initial brain tumors is these.

Improving patient outcomes requires early and quick diagnosis because it allows for targeted and timely interventions. A lot of current diagnostic methods, like biopsies and histopathological analyses, take a long time and are very invasive. They also have a high risk of causing disease and death. Using medical imaging and machine learning to improve the diagnosis and treatment of brain tumors is becoming more common. In this study, we presented a novel approach to use imaging features to predict diffuse gliomas of the adult type. In order to evaluate imaging data gathered from image sources, this study used a specially created MnasNet deep learning model. An upgraded Single Candidate Optimizer (ASCO) was used to enhance MnasNet. An external dataset showed that the model did better than the best methods, which proved that it worked. The results show that the suggested model does a better job of accurately predicting adult-type diffuse glioma with high sensitivity and specificity than current methods. This study emphasizes the potential of deep learning techniques to improve the diagnosis and treatment of brain tumors. Adult-type diffuse glioma can be diagnosed and treated more easily with the help of the recommended non-invasive technique. The model has the potential to increase diagnostic precision and reduce the need for invasive diagnostic procedures, which makes the clinical results of this study noteworthy.

## Data Availability

The data presented in the study are deposited in the Nagoya University Hospital clinical database and is available upon reasonable request from the corresponding author.

## Appendix I. Algorithm validation.

The trustworthiness of the Advanced Single Candidate Optimizer (ASCO) underwent a comprehensive validation process, which involved assessments of its performance against twenty-three valued benchmarks and comparisons with five prominent competitors (18).

The comparative algorithms include Gaining-Sharing Knowledge-based algorithm (GSK) (19), War Strategy Optimization (WSO) (20), Atom Search Optimization (ASO) (21), Butterfly Optimization Algorithm (BOA) (22), and World Cup Optimization (WCO) (23). **Table 1** presents the specified parameter values evaluated for the algorithms, thereby confirming a thorough evaluation of the accuracy of the ASCO. **Table 3** illustrates the specified parameter values evaluated for the algorithms.

**Table 3:** The specified parameter values evaluated for the algorithms confirming a thorough evaluation of the accuracy of the ASCO.

Algorithm	Set the parameter and specify its value
GSK (19)	$k_{f}$=0.5, $k_{r}\;$=0.4, $k_{r_{j}}$=0.2, $k_{r_{s}}$=0.8
WSO (20)	w=0.1, a=0.6, d=0.4, s=0.5
ASO (21)	α=60, β=0.1
BOA (22)	P=0.9, α*=*0.1, c=0.05
WCO (23)	Play off=0.05, ac=0.3

By comparing the proposed algorithm with the other algorithms on a diverse set of thirty-dimensional benchmark functions, classified into three distinct groups: mono-modal (F1-F7), multi-modal (F8-F13), and steady-dimension (F14-F23). The main objective was to identify the algorithm that could efficiently locate the minimum values across these twenty-three functions.

To guarantee a fair comparison, each algorithm’s performance was assessed based on its ability to provide the minimum result, with the most effective algorithm being the one that consistently achieved the lowest values. To evaluate the algorithms’ performance, two key metrics including Standard Deviation (SD) and Mean Value have been employed.

To keep a level playing field, a maximum of 200 iterations and a population size of 60 have been set for the algorithms. Furthermore, to enhance the reliability of the results, we repeated the optimization process 20 times. **Table 4** indicates the comparative analysis of the optimization for the proposed ASCO toward the others.

**Table 4:** Comparative analysis of the ASCO toward the others.

Function	Mean/StD	GSK	WSO	WCO	ASO	BOA	ASCO
F1	Mean	1.63E-08	1.97E-07	7.59E-18	7.16E-18	5.88E-18	5.42E-18
	StD	1.26E-04	1.79E-04	7.46E-10	5.90E-10	6.12E-10	5.56E-10
F2	Mean	1.28E-04	1.88E+01	1.04E-08	8.78E-09	8.58E-09	8.03E-09
	StD	1.79E-02	2.68E+00	3.39E-05	2.33E-05	2.15E-05	2.03E-05
F3	Mean	5.30E+00	5.17E+01	4.32E+00	3.22E+00	3.12E+00	2.87E+00
	StD	1.10E+00	4.71E+00	6.73E-01	6.19E-01	4.16E-01	3.91E-01
F4	Mean	2.08E-01	3.98E+00	2.21E-04	2.41E-04	1.99E-04	1.83E-04
	StD	9.07E-02	3.26E-01	2.32E-02	2.29E-02	2.03E-02	1.87E-02
F5	Mean	1.55E+01	4.95E+01	7.84E+00	7.83E+00	5.67E+00	5.35E+00
	StD	3.80E+00	6.07E+00	1.21E+00	1.33E+00	1.10E+00	1.06E+00
F6	Mean	1.33E-08	1.34E-07	2.74E-11	2.24E-11	1.84E-11	1.83E-11
	StD	1.04E-04	1.49E-04	7.49E-10	7.97E-10	6.45E-10	5.89E-10
F7	Mean	2.88E-02	1.58E-02	2.47E-03	2.64E-03	2.29E-03	2.09E-03
	StD	6.43E-02	5.15E-02	1.98E-02	1.72E-02	1.26E-02	1.16E-02
F8	Mean	-2.59E+03	-2.62E+03	-2.13E+03	-2.21E+03	-2.39E+03	-2.52E+03
	StD	1.40E+01	9.20E+00	2.95E+00	2.65E+00	2.46E+00	2.43E+00
F9	Mean	1.65E+01	2.54E+01	4.69E+00	4.42E+00	3.44E+00	3.13E+00
	StD	8.84E-01	1.17E+00	2.86E-01	2.60E-01	2.92E-01	2.38E-01
F10	Mean	1.80E-02	8.50E-01	1.61E-04	1.75E-04	1.54E-04	1.47E-04
	StD	1.39E-01	2.52E-01	4.06E-06	3.76E-06	3.32E-06	3.02E-06
F11	Mean	2.26E-03	7.24E-02	1.24E-03	1.09E-03	1.16E-03	9.83E-04
	StD	2.22E-02	2.38E-01	1.78E-02	1.14E-02	1.15E-02	1.06E-02
F12	Mean	3.23E-03	1.98E-01	1.67E-03	1.61E-03	1.37E-03	1.24E-03
	StD	9.26E-02	5.48E-01	4.44E-02	3.53E-02	3.33E-02	3.12E-02
F13	Mean	1.05E-03	6.44E-04	2.16E-04	2.09E-04	1.66E-04	1.52E-04
	StD	2.88E-02	2.38E-02	1.52E-02	1.11E-02	1.05E-02	1.02E-02
F14	Mean	1.94E+00	8.53E-01	3.24E-01	3.66E-01	3.11E-01	2.93E-01
	StD	4.56E-01	3.30E-01	3.66E-01	3.42E-01	2.75E-01	2.65E-01
F15	Mean	3.61E-04	4.36E-04	2.49E-04	2.23E-04	2.19E-04	1.97E-04
	StD	8.27E-03	3.05E-02	5.92E-03	6.63E-03	5.87E-03	5.49E-03
F16	Mean	-2.24E-01	-4.40E-01	-4.04E-01	-4.51E-01	-4.68E-01	-4.97E-01
	StD	7.53E-05	9.17E-05	8.74E-05	8.25E-05	6.51E-05	5.87E-05
F17	Mean	6.73E-02	1.93E-01	7.67E-02	7.31E-02	7.12E-02	6.60E-02
	StD	1.10E-08	5.41E-08	9.24E-09	7.57E-09	8.05E-09	6.88E-09
F18	Mean	1.18E+00	7.88E-01	6.69E-01	4.77E-01	4.17E-01	4.06E-01
	StD	1.37E-08	1.79E-07	6.46E-09	7.03E-09	5.81E-09	5.55E-09
F19	Mean	-8.04E-01	-1.84E+00	-1.18E+00	-2.17E+00	-1.77E+00	-2.03E+00
	StD	3.95E-08	5.95E-08	1.37E-08	1.29E-08	1.11E-08	1.06E-08
F20	Mean	-1.11E+00	-1.25E+00	-7.22E-01	-1.44E+00	-1.44E+00	-1.36E+00
	StD	1.05E-01	1.12E-01	1.07E-08	1.14E-08	8.83E-09	8.60E-09
F21	Mean	-1.62E+00	-2.61E+00	-2.66E+00	-3.40E+00	-3.02E+00	-3.13E+00
	StD	7.65E-01	6.46E-01	7.36E-01	6.30E-01	4.85E-01	4.63E-01
F22	Mean	-2.99E+00	-2.50E+00	-4.47E+00	-4.70E+00	-4.81E+00	-4.70E+00
	StD	5.07E-01	5.04E-01	2.34E-01	1.80E-01	1.51E-01	1.47E-01
F23	Mean	-2.02E+00	-1.36E+00	-3.55E+00	-4.30E+00	-3.48E+00	-3.88E+00
	StD	7.07E-01	3.77E-01	3.12E-01	2.53E-01	1.78E-01	1.74E-01

The results show the mean and standard deviation values for 23 benchmark functions, categorized into three groups: mono-modal (F1-F7), multi-modal (F8-F13), and steady-dimension (F14-F23). Overall, the results indicate that ASCO outperforms the other algorithms in terms of mean and standard deviation values.
